# Characterization of α-Glucosidases From *Lutzomyia longipalpis* Reveals Independent Hydrolysis Systems for Plant or Blood Sugars

**DOI:** 10.3389/fphys.2019.00248

**Published:** 2019-04-10

**Authors:** Samara G. da Costa, Paul Bates, Rod Dillon, Fernando Ariel Genta

**Affiliations:** ^1^Laboratory of Insect Biochemistry and Physiology, Oswaldo Cruz Institute, Fundação Oswaldo Cruz, Rio de Janeiro, Brazil; ^2^Division of Biomedical and Life Sciences, Faculty of Health and Medicine, Lancaster University, Lancaster, United Kingdom; ^3^National Institute of Science and Technology for Molecular Entomology, Rio de Janeiro, Brazil

**Keywords:** *Lutzomyia longipalpis*, digestion, sugar, sucrase, α-glucosidase, glucoside hydrolase

## Abstract

*Lutzomyia longipalpis* is the main vector of *Leishmania infantum* and exploits different food sources during development. Adults have a diet rich in sugars, and females also feed on blood. The sugar diet is essential for maintaining longevity, infection, and Leishmaniasis transmission. Carbohydrases, including α-glucosidases, are the main enzymes involved in the digestion of sugars. In this context, we studied the modulation of α-glucosidase activities in different feeding conditions and compartments of *Lutzomyia longipalpis* females, in order to characterize in detail their roles in the physiology of this insect. All tissues showed activity against MUαGlu and sucrose, with highest activities in the midgut and crop. Activity was 1,000 times higher on sucrose than on MUαGlu. Basal activities were observed in non-fed insects; blood feeding induced activity in the midgut contents, and sugar feeding modulated activity in midgut tissues. α-glucosidase activity changed after female exposure to different sugar concentrations or moieties. α-glucosidases from different tissues showed different biochemical properties, with an optimum pH around 7.0–8.0 and ***K***_M_ between 0.37 and 4.7 mM, when MUαGlu was used as substrate. Using sucrose as substrate, the optimum pH was around 6.0, and ***K***_M_ ranges between 11 and 800 mM. Enzymes from the crop and midgut tissues showed inhibition in high substrate concentrations (sucrose), with ***K***_I_ ranging from 39 to 400 mM, which explains the high ***K***_M_ values found. Chromatographic profiles confirmed that different α-glucosidases are been produced in *L. longipalpis* in different physiological contexts, with the distinction of at least four α-glucosidases. The results suggest that some of these enzymes are involved in different metabolic processes, like digestion of plant sugars, digestion of blood glycoproteins or glycolipids, and mobilization of energetic storages during starvation.

## Introduction

There are approximately 900 species of phlebotomines and about 98 of these species are considered of medical importance, being vectors of diseases such as visceral and cutaneous leishmaniasis, bartonellosis, besides transmitting other trypanosomatids and arboviruses (Sherlock, 2003; Bates et al., 2015; Oryan and Akbari, 2016; WHO, 2017).

Leishmaniasis are caused by more than 20 species of *Leishmania* (Akhoundi et al., 2016). The resulting type of infection depends on the infecting *Leishmania* species. The most common form of the disease is cutaneous leishmaniasis. In Brazil *Leishmania braziliensis* is the prevalent parasite in man causing cutaneous infections (Vianna, 1911, 1914; Gontijo and de Carvalho, 2003) and *Leishmania infantum* is the etiologic agent of visceral leishmaniasis, the most severe form (Penna, 1934; Lainson and Shaw, 1992). The several *Leishmania* species are transmitted by different phlebotomines. The main vector of *Leishmania infantum*, the etiologic agent of visceral leishmaniasis in the Americas, is the sand fly *Lutzomyia longipalpis* (Soares and Turco, 2003).

Phlebotomines explore different food sources in their larval or adult phase. Larvae are detritivore animals and grow in decaying materials, mainly plant tissues or animal feces (Moraes et al., 2012, 2014). In adults, sugar meals are essential for their energy requirements, and they feed on plant tissues, nectar of flowers and secretions produced by aphids and coccids (Killick-Kendrick and Killick-Kendrick, 1987; Moore et al., 1987; MacVicker et al., 1990; Cameron et al., 1995; Junnila et al., 2011). Females also feed on blood for egg maturation (Schlein, 1986). In this respect, these insects have a diet rich in sugars such as sucrose, maltose, trehalose, and melezitose, obtained from sugar meals and glycolipids, and glycoproteins, obtained from blood meals, which reinforces the importance the study of carbohydrases in their digestion process. The adult feeding habit of *L. longipalpis* has been the focus of several studies, since the parasite *Leishmania* is ingested in its amastigote form and transmitted during the blood supply of adult females in the metacyclic form Kamhawi (2006). Besides this, carbohydrases were described as important during the development of the *Leishmania* parasite inside the phlebotomine gut (Schlein, 1986; Pimenta et al., 1992; Dillon and El-Kordy, 1997). The sugars ingested by sand flies are hydrolyzed by glycosidases in small sugars units’, which can be absorbed by the parasites and assist their development (Gontijo et al., 1996).

α-Glucosidases are an important group of enzymes that are specialized in sugar digestion. The starch found in leaves is hydrolyzed by α-amylase to maltose, which is then cleaved to glucose by α-glucosidases (Dixon and Webb, 1979). In the same way, these enzymes are involved in the hydrolysis of sucrose acquired from the sap. The energy obtained by the hydrolysis of these sugars is a crucial factor for insect development and maintenance of a sufficient lifetime for infection with *Leishmania* and its subsequent transmission. This enzyme may also participate in the final digestion of blood glycoproteins and glycolipids.

In general, α-glucosidases (EC 3.2.1.20) are typical exo-type hydrolases and catalyze the hydrolysis of α-glucosidic linkages releasing D-glucose residues from the non-reducing end of α aryl glycosides, disaccharides, and oligosaccharides with varying efficiency (Chiba, 1997). Based on substrate specificity, α-glucosidases can be classified into three types and also can be divided in families I and II based on the primary structure. Insects α-glucosidases are classified in family I and they have no similarity with α-glucosidases from mammals, plants, and fungi, classified in family II (Chiba, 1997). α-Glucosidases from family I have four conserved regions, including the active site. These conserved regions are also found in α-amylases, but they do not share any sequence similarity.

Glycoside hydrolases (GHs), the enzyme group to which α-glucosidases belong, are also classified based on the secondary and tertiary structure in more than 100 different families. α-glucosidases were already described as members of families 4, 13, 31, 63, 97, or 122 of the glycoside hydrolases (GHF). In insects, these enzymes are found in families 13 and 31 (Cantarel et al., 2009).

The detailed description of the specificity, function, and structure of the α-glucosidases of *L. longipalpis* may provide the basis for the development of new strategies to control these insects and block the transmission of the disease, due to the importance of this enzyme in the digestion process in these insects and in the relationship between host and parasite.

A few works have described α-glucosidases activities in Phlebotomine sand flies (Samie et al., 1990; Dillon and El-Kordy, 1997; Jacobson et al., 2001, 2007) and an α-glucosidase in *L. longipalpis* was previously described (Gontijo et al., 1998). In this work, we studied the modulation of α-glucosidase activities in different feeding conditions and compartments of *L. longipalpis* females, in order to characterize in greater detail its importance for the physiology of this insect. The α-glucosidase assays were adjusted to the characteristics of sand fly enzymes, allowing us to detect activities that were not previously described. At least four different α-glucosidases with distinct biochemical properties were found, as constitutive or induced, in different feeding conditions. They appear to be involved in different metabolic processes, like digestion of plant sugars, digestion of blood glycoproteins or glycolipids, and mobilization of energetic storages during starvation.

## Materials and Methods

### Chemicals

The reagents sucrose, fructose, maltose, glucose, and methylumbelliferyl-α-glucopyranoside (MUαGlu) were purchased from Sigma-Aldrich Company (St. Louis, MO, United States). Pierce^TM^ BCA Protein Assay Kit was obtained from Thermo Fisher Scientific (Waltham, MA, United States). Glucose oxidase (GOD) was acquired from Bioclin (Minas Gerais, Brazil). Other reagents used in this work were analytical grade.

### Insects

All experiments were performed using insectary-reared *L. longipalpis* Jacobina strain (from Jacobina, Bahia, Brazil). Insects were kept under standard laboratory conditions under a temperature of 26°C (±2) and a relative humidity of ≥80% inside the rearing cages (Moraes et al., 2012, 2014, 2018). Adults sand flies were fed with 70% (w/v) autoclaved sucrose solution in cotton wool (unless stated differently in experiments). For oviposition females were fed on hamsters (*Mesocricetus auratus*) anesthetized with xylazine (10 mg/kg) plus ketamine (200 mg/kg). Engorged females were transferred to rearing containers with a piece of cotton wool soaked in sugar solution on it. The eggs were separated from dead females’ bodies after oviposition. All procedures involving animals were approved by the Animal Research Ethics Board of Oswaldo Cruz Institute (CEUA L-029/2016) and all experimental procedures were conducted following the biosecurity and institutional safety rules (CDC, 2013).

Recently emerged females (0–3 h) were fed with water for 5 days, or fed with 1.2 M sucrose for 10 days, or fed with sucrose in different concentrations (0.3 M, 0.6 M, and 1.2 M) for 2 days, or fed with mono or disaccharides (glucose, fructose, sucrose, and maltose) 1.2 M for 2 days. In some experiments, females were transferred from rearing containers to cages containing a Petri dish covered with parafilm and small drops of the offered sugar mixed with 50% (v/v) of blue food dye (Arcolor, São Paulo, Brazil).

Females fed with blood for experiments had no previous contact with sugar. Recently emerged females (0–3 h) were maintained for 3 days with cotton wool soaked in water before feeding on hamsters. After blood feeding, they were maintained with a piece of cotton wool soaked in water for 4 days.

### Quantification of the Volume Ingested

When different concentrations of sucrose or different sugars were offered to females, a blue food dye (Arcolor, São Paulo, Brazil) 50% (v/v) was mixed with sugar to determine the volume ingested by females (Ferreira et al., 2018). Briefly, 2 days after feeding on sugars, females were individually dissected in saline phosphate buffer (PBS) and the whole gut was homogenized in 2–10 μL of water for proper dilution. Samples were centrifuged at 10,000 × *g* for 5 min. An aliquot of 1 μL from the supernatant was used to read the absorbance at 630 nm using a NanoDrop^^®^^ (Thermo Fisher Scientific). The volume ingested was calculated from a standard curve assembled and read under the same conditions as the samples.

### Sample Preparation

Females were dissected on PBS and the midgut was individually homogenized in 20 μL of 50 mM citrate-phosphate buffer pH 6.0 and centrifuged at 20,000 × g for 10 min at 4°C. The supernatant was saved and the pellet was washed again with 20 μL of 50 mM citrate-phosphate buffer pH 6, being recentrifuged at 20,000 × *g* for 10 min at 4°C. Supernatants were pooled resulting and named “midgut contents” fraction. The pellet was suspended in 40 μL of 50 mM citrate-phosphate buffer pH 6 containing 1% (v/v) Triton X-100. After 2 h of incubation on ice, it was centrifuged at 20,000 × *g* for 10 min at 4°C. This supernatant was named “midgut tissue” fraction. When head, carcass, crop or hindgut were used for α-glucosidase assays they were individually homogenized in 40 μL of 50 mM citrate-phosphate buffer pH 6 containing 1% (v/v) Triton X-100 and incubated on ice for 2 h. These samples were centrifuged at 20,000 × *g* for 10 min at 4°C and the supernatant was used as a source of enzymes. For all samples the following protease inhibitors were mixed (final concentrations): PMSF 4 mM, E64 0.02 mM, and pepstatin 0.02 mM. When samples were used for studies of the effect of pH, substrate concentration or chromatography the samples consisted of pools obtained from 100 females.

### α-Glucosidase Assay and Protein Determination

α-Glucosidase activity was measured in individual freshly prepared samples, by continuous assay using MUαGlu as substrate and discontinuous assay using sucrose as substrate. Briefly, samples were incubated for 1 h at 30°C using 200 mM AMPSO pH 8.0 buffer and 0.1 mM MUαGlu (final concentration) (Sigma cat. no. M9766), the final volume reaction was 100 μL. The amount of 4-methylumbelliferone released was determined continuously at 355 nm excitation and 460 nm emission (Profeta et al., 2017) in a 96-well SpectraMax Gemini XPS Microplate Reader (Molecular Devices). The amount of product was calculated from a standard curve of 4-methylumbelliferone assembled and read under the same conditions as samples.

In assays with sucrose as substrate, samples were incubated for 30 min at 30°C using 200 mM citrate-phosphate pH 6.0 buffer and 200 mM sucrose (final concentration), the final volume reaction was 50 μL. Reactions were interrupted at different time intervals (0, 10, 20, and 30 min) by incubating the mixture at 99°C for 5 min. The amount of glucose released was determined according to the specifications provided by the manufacturer of the glucose mono-reagent kit (Bioclin, Brazil), based on the method described by Raabo and Terkildsen (1960). Adjustments were done for the small volumes used. Briefly, 200 μL of GOD reagent was added to 50 μL reactions and incubated for 15 min at 37°C. Plates were read at 505 nm in a 96-well SpectraMax 190 Microplate Reader (Molecular Devices). The amount of product was calculated from a standard curve of glucose assembled and read under the same conditions as samples.

Controls without substrate or samples were incubated at the same conditions used for the assay of each substrate. One unit of enzymatic activity (U) is defined as the amount of enzyme which releases 1 μmol of product/min.

Protein concentration was determined using Pierce^TM^ BCA Protein Assay Kit (Thermo Fisher Scientific) (Smith et al., 1985), following the protocol provided by the manufacturer for microplate procedures, using bovine serum ovalbumin as a standard.

### Effect of Substrate Concentration and pH on α-Glucosidase Activity

The effect of pH on enzyme activity was studied at 30°C using 0.1 mM MUαGlu or 200 mM sucrose (final concentrations) as substrates and the following buffers (200 mM): sodium citrate-citric acid (pH 3–4), sodium acetate (pH 3.7–6), citrate-phosphate (pH 3–7), MOPS (pH 6–8), EPPS (pH 7–9), AMPSO (pH 8–10), CAPS (pH 10–11). Assays were made as described in the section “α-Glucosidase Assay and Protein Determination.”

To determinate the optimum pH in tested conditions a curve was fitted according to the equation (1) described below (Segel, 1975):

(1) $$V_{\text{max app}}=\frac{V_{\text{max}}}{1+\frac{\left[H^{+}\right]}{K_{\text{es}1}}+\frac{K_{\text{es}2}}{\left[H^{+}\right]}}$$

**SCHEME 1:**
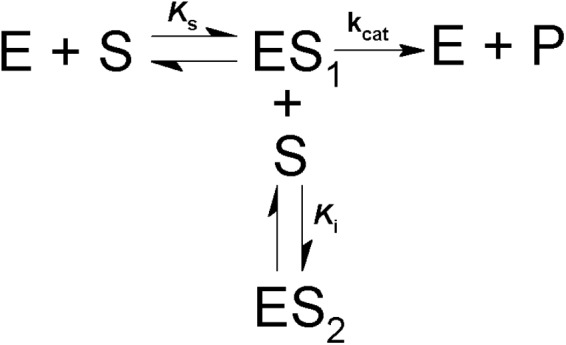
Model for simple competitive substrate inhibition. According to this, at high concentrations a second molecule of substrate (S) binds to the enzyme-substrate complex (ES_1_), forming an inactive ternary complex (ES_2_).

The effect of substrate concentrations on the activity was determined at 30°C using 200 mM AMPSO pH 8 and (0.01–2.3 mM) MUαGlu or using 200 mM citrate-phosphate pH 6.0 buffer and (10–1,400 mM) sucrose. Activity was determined depending on the substrate as described in the item “α-Glucosidase Assay and Protein Determination.” Values for apparent ***K***_M_ (***K***_M app_), apparent ***V***_max_ (***V***_max app_) and ***K***_I_ were determined using GraFit Software (GraFit version_7.0.3, Erithacus Software Limited) and the Michaelis–Menten equation as described in Segel (1975). When inhibition by high substrate concentration was detected, kinetic parameters were calculated taking into account the reaction (Scheme 1) and the equation (2) below (Cleland, 1979):

(2) $$V=\frac{V_{\text{max}}[S]}{K_{\text{M}}+[S](1+\frac{\left[S\right]}{K_{\text{i}}})}$$

### Gel Filtration Chromatography

Midgut soluble and midgut tissue fractions were applied (sample volume 0.5 mL) to a Superdex 200 10/300 GL (1 cm × 30 cm) (AKTA Purifier, GE, Uppsala, Sweden). The column was equilibrated with 50 mL of 20 mM citrate-phosphate buffer pH 6.0 for midgut content sample and 20 mM citrate-phosphate buffer pH 6.0 containing 1% Triton X-100 for midgut tissue sample. Proteins were eluted with the same buffer used to equilibrate column at 0.5 mL.min^-1^. Fractions of 0.5 mL were collected and assayed with MUαGlu and sucrose as described in the section “α-Glucosidase Assay and Protein Determination.”

### Statistical Analysis

All statistical analysis were performed using GraphPad Prism 6.0 for Windows (San Diego, CA, United States). To identify outliers the ROUT method based on the false discovery rate (FDR) was used and Q was established as 1%. To determine how far the distribution is from Gaussian in terms of asymmetry and shape the D’Agostino-Pearson Omnibus K2 normality test was used. For comparison of normally distributed data, one-way ANOVA was used followed by Tukey’s multiple comparison tests, and significance was considered when *p* < 0.05. Results are expressed as the means ± SEM.

## Results

### Distribution of α-Glucosidase Activity in Tissues Using Different Substrates

**FIGURE 1:**
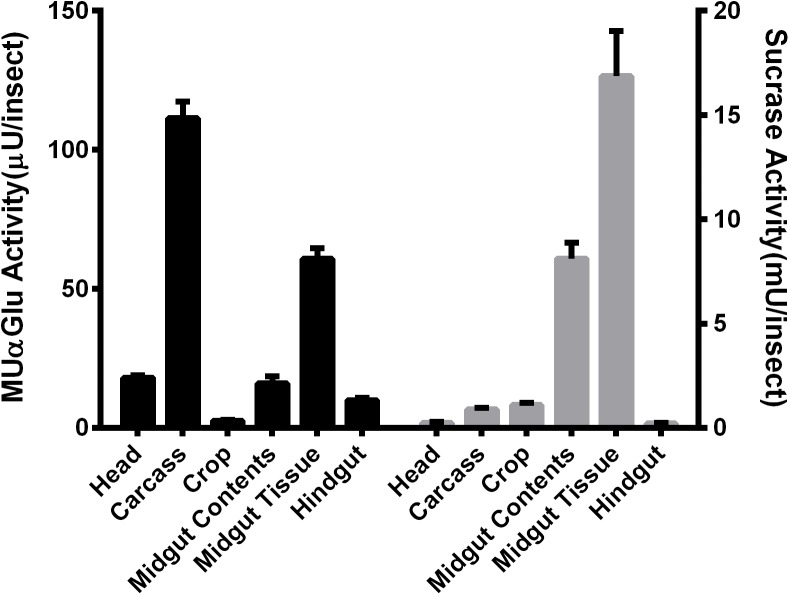
Comparison of α-glucosidase activity using as substrate MUαGlu (black bars) or sucrose (gray bars) in different tissues of recently emerged (0–3 h) *L. longipalpis* females. The results are the mean ± SEM of three biological replicates each with *n* = 12.

Different parts of *L. longipalpis* females, collected at 0–3 h after emergence, were individually assayed using MUαGlu and sucrose as substrates to see the anatomical distribution of α-glucosidase activities. Activity was detected in all tissues, but the distribution was different depending on the substrate used (Figure 1). Using MUαGlu as substrate, most activity was found in the carcass (111 ± 5 μU/insect), but the enzymes present in the midgut also showed significant activity. Using sucrose as substrate, highest activities were localized in the midgut contents (8.1 ± 0.7 mU/insect) and midgut tissues (16 ± 2 mU/insect). The results also show that the activity is much higher on sucrose than on MUαGlu, showing the preference of these enzymes for the natural substrate. In the case of crop and midgut contents, activity was 2,500 and 3,700 times, respectively, higher on sucrose than MUαGlu.

### Detection of α-Glucosidase Activities in the Midgut of Females Fed With Water or Sucrose

**FIGURE 2:**
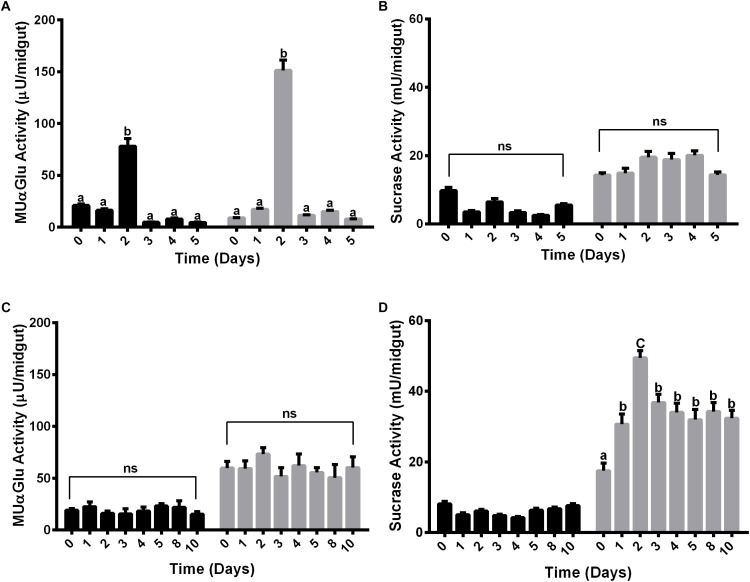
α-Glucosidase activity in different tissues of *L. longipalpis* females feeding on water **(A,B)** or 1.2 M sucrose **(C,D)**. For each feeding condition, the midgut contents (black bars) and midgut tissues (gray bars) were analyzed. Activity was determined using MUαGlu **(A,C)** or sucrose **(B,D)** as substrates. Numbers in the *x*-axis represent the time of exposure to water or sucrose after adult emergence. The results are the mean ± SEM of three biological replicates each with *n* = 15. One-way ANOVA, followed by Tukey multiple comparison test. Different letters indicate statistically significant different groups, *p* < 0.05. ns, non-significant difference.

The activity of α-glucosidase was measured in the midgut contents and midgut tissues of females fed with water or sucrose for 5 or 10 days, respectively. Females were initially fed with water only to verify if there was some sucrase induction associated with age (Figure 2A,B). Interestingly, an induction happened for both midgut contents and midgut tissues after 2 days for the activity against MUαGlu (Figure 2A). However, no induction was identified for activity against sucrose in females fed only with water. Due to the increasing mortality of insects, we were able to follow these activities only until 5 days.

When feeding females with 1.2 M sucrose for 10 days, we observed different expression profiles for the α-glucosidase activity when we compare the two substrates (Figure 2C,D). In the midgut contents no induction was observed during the 10 days of feeding with sucrose for both substrates (Figure 2C,D), although for midgut tissue there was an induction of activity against sucrose, with a peak after 2 days of feeding with sugar (Figure 2D). A significant increase of 2.8 times in the activity can be observed when comparing recently emerged females (17 ± 2 mU/midgut) to females fed with 1.2 M sucrose (49 ± 2 mU/midgut). After 3 days, the activity decreases but does not return to basal levels, suggesting that the sucrase activity induction occurred due to the presence of sugar meal (Figure 2B). However, there was no induction in the midgut tissue when we followed activity against MUαGlu (Figure 2C).

**FIGURE 3:**
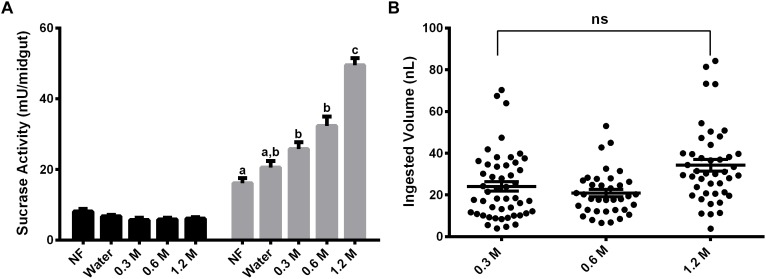
Effect of feeding *L. longipalpis* females with different sucrose concentrations for 2 days, on the α-glucosidase activity. For each feeding condition midgut contents (black bars) and midgut tissues (gray bars) were analyzed **(A)**. The volume ingested in each sucrose concentration was also examined **(B)**. The *x*-axis represents the concentration of sugar used to feed the females. Activity was measured using sucrose as substrate. NF – Recently emerged females (0–3 h), Water – females fed with only water for 2 days. The results are the mean ± SEM of three biological replicates each with *n* = 15. One-way ANOVA, followed by Tukey multiple comparison test. Different letters indicate statistically significant different groups, *p* < 0.05. ns, non-significant difference.

Females were also fed with different concentrations of sucrose (0.3 M, 0.6 M, and 1.2 M) for 2 days and the activity against sucrose was measured, to verify if the expression observed has any relation with the sugar concentration in the meal (Figure 3A). The activity in the midgut contents was not induced by any of the sucrose concentrations tested (Figure 3A). Nevertheless, in the midgut tissue we observed a dose–response relationship between the sucrase activities and sugar concentrations in the sugar meal, from 0.3 to 1.2 M. This is not a consequence of higher ingestion rates, as an equal meal volume was ingested for all sugars concentrations (Figure 3B).

### Induction of α-Glucosidase Activity in the Midgut of Females Fed With Mono or Disaccharides

**FIGURE 4:**
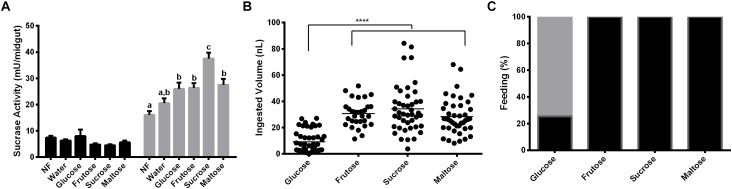
Effect of feeding *L. longipalpis* females with different 1.2 M mono and disaccharides for 2 days on α-glucosidase activity. For each feeding condition midgut contents (black bars) and midgut tissues (gray bars) were analyzed **(A)**. The volume of mono or disaccharide solution ingested was also examined **(B)**. Representation of the percentage of females that ingested the mono or disaccharide solution. Fed females (black), unfed females (gray) **(C)**. Activity was measured using sucrose as substrate. NF – Recently emerged females (0–3 h), Water – females fed only with water for 2 days. The results are the mean ± SEM of three biological replicates each with *n* = 15. One-way ANOVA, followed by Tukey multiple comparison test. Different letters indicate statistically significant different groups, *p* < 0.05. ^∗∗∗∗^ indicates statistically significant differences in ingested volumes for different sugars, *p* < 0.001.

Females were fed with glucose, fructose, sucrose or maltose for 2 days. α-Glucosidase activity was measured with sucrose, to understand if the sucrase activity induction relies on the type of sugar provided. Results demonstrated that after 2 days of feeding on glucose, fructose or maltose, the activity of α-glucosidase was higher than the observed in recently emerged females, but not statistically different from the observed in females that were fed on water for 2 days (Figure 4A). However, feeding with sucrose resulted in a significant higher sucrase activity when compared to all groups mentioned above (Figure 4A). Figure 4B demonstrates that there is no significant difference in the volume ingested by females feeding of fructose, sucrose, and maltose (32 ± 2; 34 ± 2; 30 ± 2 ηL per individual, respectively), neither in the percentage of insect that ingests sugar (100% in all these cases, Figure 4C). However, just a small percentage of females (29%) can feed on glucose (Figure 4C), with a significantly smaller mean volume in the ingested sugar meal (9 ± 1 ηL; Figure 4B).

### Detection of α-Glucosidase Activity in the Midgut of Females Fed With Blood

**FIGURE 5:**
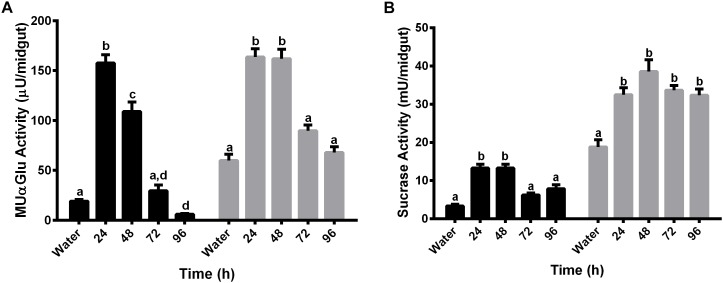
α-glucosidase activity in different tissues of *L. longipalpis* females after feeding with blood. Activity was determined in midgut contents (black bars) and midgut tissues (gray bars) using MUαGlu **(A)** or sucrose **(B)** as substrates. Numbers in the *x*-axis represent the time elapsed after ingestion of blood. Water – females fed only with water for 3 days. The results are the mean ± SEM of three biological replicates each with *n* = 15. One-way ANOVA, followed by Tukey multiple comparison test. Different letters indicate statistically significant different groups, *p* < 0.05.

We decided to test if α-glucosidase activities are also involved in blood digestion. For this, activity was measured with MUαGlu and sucrose as substrates, in females starved for 3 days (water-mantained), and in females fed with blood, every 24 h following feeding during 4 days (24, 48, 72, and 96 h). Activities were measured in midgut contents and midgut tissues (Figure 5A,B).

Results showed induction of both activities, against sucrose or MuαGlu, in all fractions (midgut contents and midgut tissues) 24 h after blood feeding. The activity observed in the midgut contents after blood feeding seems to be different from the previous activity observed after sugar feeding, as we observed induction by blood and no induction when females are fed with 1.2 M sucrose (Figure 2, 5). In the case of midgut tissues, the pattern of induction is also different, as we observed an increase in activity against MUαGlu after 24 h, wich was absent in females fed with sucrose (Figure 2A, 5A).

### Determination of α-Glucosidases Kinetic Properties: Effect of pH and Substrate Concentration on Enzyme Activities

Because we observed different expression patterns for the α-glucosidase activities when comparing the midgut contents to the midgut tissue using different substrates, we decided to measure the kinetic parameters of each activity to observe if these activities also differ in their molecular properties. The effect of pH and substrate concentration in α-glucosidase activity was determined in samples obtained from recently emerged females (non-fed, NF), females fed with 1.2 M sucrose for 2 days (SF) and females 2 days after feeding on blood (BF), using sucrose and MUαGlu as substrates.

**Table 1:** pH optimum of α-glucosidases obtained from different samples of *L. longipalpis* females under different physiological conditions, using MUαGlu or sucrose as substrates.

Sample	MUαGlu			Sucrose		
	Non-fed	Sucrose-fed	Blood-fed	Non-fed	Sucrose-fed	Blood-fed
Midgut content	7.5 ± 0.2	7.4 ± 0.3	7.4 ± 0.2	6.4 ± 0.9	6.3 ± 0.6	6.7 ± 0.2
Midgut tissue	7.0 ± 0.2	6.8 ± 0.1	7.3 ± 0.2	6.3 ± 0.4	6.5 ± 0.1	6.5 ± 0.2
Carcass	7.4 ± 0.2	7.4 ± 0.1	7.7 ± 0.2	6.2 ± 0.2	6.4 ± 0.2	6.3 ± 0.1

The results are summarized in Table 1. Enzymes acting on MUαGlu have maximum activity in neutral to basic pH, with optimum pH around 7.5 and significant activities between pH 6 and 8.0. Using sucrose as substrate a more acidic optimum pH was found, around 6.5, with significant activities between pH 6 and 7 with a substantial decrease in activity at pH 8.0. When comparing samples from the same type of sample, similar profiles were obtained under different physiological conditions (Supplementary Figures 1, 2).

**Table 2:** Kinetic parameters of α-glucosidase activities obtained from different samples of *L. longipalpis* females under different physiological conditions, using sucrose as substrate.

	Sucrose											
	Non-fed				Sucrose-fed				Blood-fed			
Sample	*V*_max app_ (mU/Fleb)	Protein (μg/Fleb)	*K*_M app_ (mM)	*K*_I_ (mM)	*V*_max app_ (mU/Fleb)	Protein (μg/Fleb)	*K*_M app_ (mM)	*K*_I_ (mM)	*V*_max app_ (mU/Fleb)	Protein (μg/Fleb)	*K*_M app_ (mM)	*K*_I_ (mM)
Crop	4.6 ± 0.6	2.5 ± 0.5	210 ± 40	150 ± 30	0.359 ± 0.007	2.7 ± 0.2	11 ± 1	–	0.58 ± 0.06	5.6 ± 0.7	110 ± 20	400 ± 100
Midgut content	5.5 ± 0.1	5.3 ± 0	44 ± 7	–	2.22 ± 0.07	5.7 ± 0.6	51 ± 6	–	11.1 ± 0.1	258 ± 7	20 ± 1	–
Midgut tissue	40 ± 10	10 ± 1	170 ± 60	180 ± 60	150 ± 20	8 ± 1	800 ± 100	39 ± 7	50 ± 10	337 ± 3	400 ± 100	70 ± 20

**Table 3:** Kinetic parameters of α-glucosidase activities obtained from different samples of *L. longipalpis* females under different physiological conditions, using MUαGlu as substrate.

	MUαGlu								
	Non-fed			Sucrose-fed			Blood-fed		
Sample	*V*_max app_ (μU/Fleb)	Protein (μg/Fleb)	*K*_M app_ (mM)	*V*_max app_ (μU/Fleb)	Protein (μg/Fleb)	*K*_M app_ (mM)	*V*_max app_ (μU/Fleb)	Protein (μg/Fleb)	*K*_M app_ (mM)
Crop	90 ± 7	2.5 ± 0.5	2.1 ± 0.2	52 ± 2	2.7 ± 0.2	0.48 ± 0.06	16 ± 1	5.6 ± 0.7	0.9 ± 0.1
Midgut content	390 ± 20	2.76 ± 0.04	0.7 ± 0.1	266 ± 7	3.8 ± 0.3	0.37 ± 0.03	830 ± 10	258 ± 7	0.41 ± 0.02
Midgut tissue	3000 ± 200	10 ± 1	4.7 ± 0.4	1400 ± 100	6.2 ± 0.5	1.1 ± 0.1	1150 ± 40	337 ± 3	0.50 ± 0.05

The kinetic properties of α-glucosidases from *L. longipalpis* in different physiological conditions are described in Tables 2, 3. Activities from the same tissue under different physiological conditions showed different substrate affinities. The kinetic parameters were also different when comparing the samples to each other in the same physiological condition.

When sucrose was used as the substrate, the ***K***_mapp_ values for all samples varied according to the condition tested. The highest affinity was found for samples of the crop from sugar-fed females and midgut content from blood-fed females. ***K***_mapp_ for midgut content changed with a significant reduction when females were fed with blood compared to non-fed females or females fed with sucrose. In the opposite side, it is important to note the high ***K***_mapp_ values found for midgut tissue for sugar and blood-fed females. As expected ***V***_max app_ values were highest for the midgut tissue, with specific activities of 4 mU/μg protein for non-fed females and 18.7 mU/μg protein for sugar-fed females.

For MUαGlu, the lowest ***K***_mapp_ was found for the midgut contents in sugar- and blood-fed insects. In the midgut contents, the ***V***_max app_ was 3X higher in blood-fed females when comparing to sugar-fed females, but the affinity for the substrate was practically unaffected. For the midgut tissue, the ***V***_max app_ was the highest found for non-fed females (300 μU/μg protein), with a specific activity 2× higher than the observed in midgut content (141.30 μU/μg protein). The crop had the lowest ***V***_max app_ in all conditions, the lowest activity being found in the crop of blood-fed females (2.8 μU/μg protein).

**FIGURE 6:**
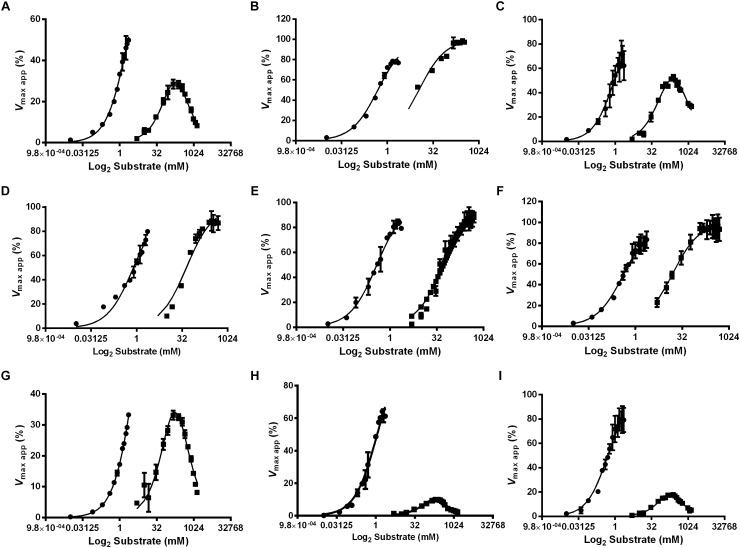
Effect of the substrate concentration on α-glucosidase activity in different feeding conditions and tissues of *L. longipalpis* females. Samples were obtained from recently emerged females (0–3 h) **(A,D,G)**, females fed with 1.2 M sucrose for 2 days **(B,E,H)** and females after 2 days of blood feeding **(C,F,I)**. For each feeding condition, crop **(A–C)**, midgut contents **(D–F)** and midgut tissue homogenates **(G–I)** were analyzed. MUαGlu (dark circles) and sucrose (dark squares) were used as substrates. The points are experimental results and lines were calculated based on constants computed with the software Graphit using the least-squares method. Note the inhibition by excess of sucrose as substrate. ***V*_max app_** (%) was calculated using experimental results compared to values found using the Graphit software (described in Tables 2, 3).

**FIGURE 7:**
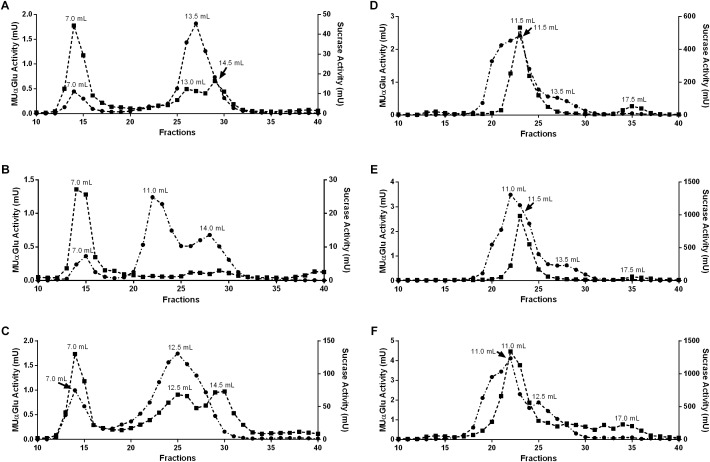
Gel filtration chromatographies (Superdex 200 column, AKTA-FPLC) of the midgut contents **(A–C)** or midgut tissue samples **(D–F)** of *L. longipalpis* females. Samples were obtained from recently emerged females (0–3 h) **(A,D)**, females fed with 1.2 M sucrose for 2 days **(B,E)** and females after 2 days of blood feeding **(C,F)**. Activity was monitored using MUαGlu (dark circles) and sucrose (dark squares) as substrates. The elution volumes of the activity peaks are indicated in the figure.

We also observed that enzymes for all tissues and feeding conditions showed classical Michaelis–Menten kinetics when MUαGlu was used as substrate but, the sucrase activity present in the crop and midgut tissue was inhibited in high sucrose concentrations, except in the crop for sucrose-fed females (Table 2 and Figure 6). Using midgut tissue preparations, the sucrase activity started to be inhibited in the presence of small concentrations of sugar, and the ***K***_i_ value found is similar or smaller than the ***K***_mapp_ for the substrate.

### Chromatographic Analysis

To further clarify if the different induction profiles and biochemical properties found in different tissues and physiological conditions correspond to α-glucosidases with different molecular properties, we subjected samples of midgut contents and tissues to gel filtration chromatographies. Representative chromatograms for all samples tested are displayed in Figure 7.

For the midgut contents we observed different peak profiles when considering the feeding conditions and substrates used (Figure 7A–C). Two major peaks with activity against MUαGlu can be identified for non-fed (7 and 13.5 mL) and blood-fed females (7 and 12.5 mL), and the activity of the first peak almost doubles in samples from blood-fed females. It is important to point the presence of a third peak in samples from sugar-fed females (7, 11, and 14 mL). When the chromatographic fractions were analyzed with sucrose as substrate different profiles are obtained, with three activity peaks for non-fed and blood-fed females, with higher activity in the last condition. For sugar-fed females only the first peak (7 mL) could be identified. The first peak had the same retention time as the ones identified with MUαGlu, so this probably corresponds to an enzyme with activity on both substrates.

For midgut tissue samples (Figure 7D–F), a major peak was identified with retention volume around 11 mL in all conditions, with significant activity against both substrates used. Using MUαGlu a minor peak appears around 13.5 mL retention volume and for sucrose at 17.5 mL. For sugar-fed and blood-fed females the activity was higher than the activities found for non-fed females, as expected. For the midgut tissue samples Triton X-100 was added to the running buffer as we were probably working with proteins solubilized from the membrane fraction. This certainly resulted in a lower resolution, since the Triton X-100 theoretically adds a molecular mass of about 90 kDa to the solubilized proteins, corresponding to the detergent micelle.

**Table 4:** Recovery of activity from peaks obtained after Gel filtration chromatographies (Superdex 200 column, AKTA-FPLC) of the midgut contents or midgut tissue samples of *L. longipalpis* females.

			Elution volume (mL)	Recovery MUαGlu activity (%)	Recovery sucrose activity (%)
Midgut content	Non-fed	Peak 1	7	49	15
		Peak 2	13	–	6
		Peak 3	13.5	351	–
		Peak 4	14.5	–	5
	Sugar-fed	Peak 1	7.5	48	13
		Peak 2	11	277	–
		Peak 3	14	173	–
	Blood-fed	Peak 1	7	30	16
		Peak 2	12.5	123	19
		Peak 3	14.5	–	10

Midgut tissue	Non-fed	Peak 1	11.5	198	80
		Peak 2	13.5	35	–
		Peak 3	17.5	–	9
	Sugar-fed	Peak 1	11	201	–
		Peak 2	11.5	–	47
		Peak 3	13.5	38	–
		Peak 4	17.5		4
	Blood-fed	Peak 1	11	115	291
		Peak 2	12.5	35	–
		Peak 3	17	–	43

*Note the elution volumes of different peaks depending on tissue and feeding condition tested.*

The activity recovered after chromatography was calculated (Table 4) and surprisingly activity against MUαGlu after chromatography was 1.5 (midgut tissue from blood-fed females) to almost 5 × (midgut content from sugar-fed females) higher than the activity quantified in samples initially applied on gel filtration. This probably suggests that an inhibitor was removed during chromatography. For sucrase activity, the recovery was higher than initial activity only for midgut tissue samples from blood-fed females.

## Discussion

Sugar meals are essential for the energy requirements of sand flies. These insects have a diet rich in sugars, glycolipids, and glycoproteins, which makes important to study carbohydrases involved in their digestion process. In this work, we demonstrate the presence of α-glucosidase activity in all tissues (head, carcass, crop, midgut, and hindgut) of *L. longipalpis* unfed females, using two different substrates (Figure 1). The α-glucosidase activity described in the crop might represent a salivary activity, a intracelular or even an activity provenient from microbiota, this compartment is part of the anterior gut and due to the ectodermal origin, crop is not capable of secreting enzymes. The distribution of the activity varied with the type of substrate used. Higher activities were found for the natural substrate sucrose, with more than 1,000× higher activities when compared to MUαGlu. This demonstrates the specialization of these enzymes for sucrose digestion. Jacobson et al. (2007) showed that the composition of sugar-degrading enzymes in the midgut of *Phlebotomus papatasi* sand flies depends on the ecological habitat they were collected, corresponding to different sugar sources. This correlates with the specialization of α-glucosidase described here in the digestion of sucrose.

The presence of carbohydrases has been reported by different groups in different sand fly species. Samie et al. (1990) analyzed the incubation of head and thorax homogenates from *P. papatasi* with sugar solutions and detected monosaccharides by HPLC analysis. They suggested these products were generated by the action of glycosidases from salivary glands and that they are mixed with the sugar meal in the crop. For mosquitoes, it is accepted that sugar digestion occurs in the crop by α-glucosidases produced by the salivary glands (James et al., 1989; Marinotti and James, 1990; Marinotti et al., 1996). Jacobson et al. (2001) described the α-glucosidase activity in the gut and salivary glands of unfed *P. papatasi.* α-glucosidase activity was found in the midgut of blood-fed *Phlebotomus langeroni* females, and a small activity at homogenates of crop, but it was considered contamination from the midgut (Dillon and El-Kordy, 1997). Gontijo et al. (1998) described α-glucosidase activity only in the midgut of non-fed *L. longipalpis.* They used the substrate pNPαGlu (p-nitrophenyl-α-glucopyranoside) and sucrose at 1 mM and 25 mM final concentrations, respectively, to characterize this activity. One difference between our work and Gontijo et al. (1998), relies on the type of substrates and their final concentrations in the assays. For sucrose the ***K***_mapp_ for these enzymes is very high. In this way, to saturate the enzyme and have a measurement of activity directly proportional to the quantity of enzyme, a very high final concentration of sucrose in the assays is necessary. When we used a small final concentration of sucrose in the assays (data not shown), we were not able to identify activity in the crop and hindgut. The concentration of the sucrose as substrate used also explains the difference in the midgut activity of unfed females of 2.3 mU/midgut (Gontijo et al., 1998) to 16 mU/midgut (our work). Besides that, the membrane-bound activity related to midgut could be higher than specified in our work, during homogenization some of the enzymes could be adhered to the homogenizer used to separate midgut contents and midgut tissue fractions.

Midgut α-glucosidase activity was found in the soluble and tissue-associated form in non-fed, sugar-fed, and blood-fed females, in accordance with what has been described by Gontijo et al. (1998). They were the first to characterize a membrane-bound α-glucosidase attached to the epithelial microvilli from the midgut of *L. longipalpis*. Enzymes involved in the late stages of digestion such as exoglycosidases are usually associated with the membrane of the midgut (Terra and Ferreira, 1994).

For non-fed females, activity against MUαGlu in the midgut content and tissue was induced after 2 days of starvation (Figure 2A), so the synthesis of this α-glucosidase do not seem to be directly related to the ingestion of sucrose. Factors like hormones can regulate the production of α-glucosidases in recently emerged females (Souza-Neto et al., 2007). We suggest that this might represent the activity of α-glucosidases involved in the intracellular metabolism, like lysosomal α-glicosidases that participates in the mobilization of energetic reserves, or α-glucosidases involved in the processing of glycoproteins in the endoplasmic reticulum.

In sugar-fed females, sucrase activity in the midgut tissue was also induced after 2 days (Figure 2D). Besides that, this induction was variable depending on the sugar concentration and the type of sugar used to feed females (Figure 3). In this way, these α-glucosidases seem to be induced by sugar ingestion. After induction, this activity does not return to basal levels, when compared to non-fed females. This might reflect the dinamics of sugar digestion inside the sand fly, where part of the sugar meal is directed to the empty midgut and the rest is stored inside the crop. After the meal, small amounts of sugars are released into the midgut during the subsequent days. Jacobson and Schlein (2001) described the increase of α-glucosidase activity in the homogenates of females of *P. papatasi* that fed during the night on the plant *Capparis spinosa*, rich in sucrose and starch. Jacobson et al. (2007) analyzed α-glucosidase activity in females of *P. papatasi* from different ecological habitats, and they showed an increase in the activity measured with sucrose as substrate after feeding with 1 M sucrose and a decrease in activity when pNPαGlu was used. Dillon and El-Kordy (1997) described an activity increase in the midgut of *P. papatasi* after feeding insects with 30% (w/v) sucrose solution.

When we used different sugars to feed *L. longipalpis* females, an increase in the activity was found in the midgut tissue of insects fed with 1.2 M sucrose (Figure 4). This effect can be due to the fact that we used sucrose itself to measure the activities. If maltose had been used as substrate, we might have found a different profile, with the higher activity being detected in females that were fed on 1.2 M maltose. Different works demonstrated that the repertory of glycosidases secreted seems to match the source of sugar present in the diet of these insects (Jacobson et al., 2007; Souza-Neto et al., 2007).

Interestingly, when females were fed with blood an induction was observed in both midgut contents and tissue after 24 h, and this induction was found using MUαGlu and sucrose as substrates (Figure 5). These females had no previous contact with sugar suggesting that this induction was specifically triggered by blood ingestion. Since the induction of activity in the midgut contents was detected using both substrates, this demonstrates that an α-glucosidase with high activity against sucrose might be acting in the digestion of blood. In *P. langeroni*, a soluble α-glucosidase activity was described in the midgut after blood ingestion, being rapidly produced (after 1 h) with maximum activity 24 h after blood feeding (Dillon and El-Kordy, 1997).

It is possible that these enzymes cross the peritrophic matrix and act in the endoperitrophic space, together with other glycosidases, in the hydrolysis of blood glycoconjugates. The increase in this activity (24 h) suggests that these enzymes may be stored in the cells of the gut being secreted in response to the blood supply, with more enzymes being synthesized and secreted as needed. The blood has predominantly protein in their composition and a high proportion of glycoproteins, and their carbohydrate portions along with glycolipids are theoretically susceptible to cleavage by exoglycosidases including α-glucosidases. Fragments of glycopeptides produced by the proteolytic cleavage of trypsin and aminopeptidases are more susceptible to the action of exoglycosidases, so the increase of α-glucosidase activity may follow the pattern of protease activity. Another plausible role for soluble α-glucosidase induction is the involvement in the detoxification of heme during blood digestion, auxiliating the nucleation of hemozoin cristals, as described for α-glucosidases from *Rhodnius prolixus* (Mury et al., 2009).

In sand flies, the induction of protease activity after the blood feeding seems to reach a peak between 24 and 48 h with some variation depending on species and type of blood ingested (Borovsky and Schlein, 1987; Dillon and Lane, 1993; Ramalho-Ortigão et al., 2003). For *L. longipalpis*, according to Sant’Anna et al. (2009) and Moraes et al. (2018), trypsin activity reaches a maximum activity 48 h after blood-feeding, which is consistent with the α-glucosidase activity observed in our experiments. An additional issue that remains to be investigated is that part of the activity present in the midgut contents might be derived from microbial glycosidases.

The characterization of the activities present in different tissues of *L. longipalpis* showed that the pH of optimal activities is only slightly affected by the feeding condition or tissue localization of enzymes (Table 1). Most differences in the effect of pH on activity are related to the type of substrate used. Activity against MUαGlu showed a wider range of optimal activity, between 7 and 8 (Supplementary Figure 1). Activity against sucrose is more affected by pH changes with a more sharp profile in the adjustment curve, and optimal activity around 6.0 (Supplementary Figure 2). These results are consistent with the pH value determined in the gut of sucrose-fed females, which is approximately 6 (Gontijo et al., 1998). It is interesting to observe that when we measured the activity with sucrose, even for females digesting blood, the enzymes display a better performance under an acidic environment. In Santos et al. (2008) demonstrated that the pH in the abdominal midgut becomes alkaline during blood digestion but the thoracic midgut remained acid (pH 5.5–6.0). They also demonstrated a higher activity of α-glucosidase in the thoracic midgut in comparison to the abdominal midgut in non-fed females. All these results together suggest that different α-glucosidases are acting in the thoracic and anterior midgut in the sugar and blood digestion. For *P. langeroni* an α-glucosidase with an optimum pH of 7.5 was detected in blood-fed females, suggesting an alkaline activity induced only in blood-fed females (Dillon and El-Kordy, 1997). In this case, the substrate used was pNPαGlu, which may mymic the synthetic substrate used in our study (MUαGlu).

The results showed in our work demonstrates that *L. longipalpis* have different α-glycosidases/sucrases with different affinities and behaviors depending on substrate (Tables 2, 3). Some of them did not follow the classical Michaelis–Menten kinetics and were inhibited by high concentrations of sucrose. We showed a ***K***_Mapp_ variation from 0.37 to 4.7 mM when MUαGlu was used as substrate and from 11 to 800 mM when sucrose was the substrate. These high ***K***_Mapp_ values described for sucrose substrate suggest that these enzymes may show a huge variation in activity in a broad range of sucrose concentrations, which may be compatible with the feeding of nectars presenting sucrose in concentrations as high as 1.2 M (Heil, 2011). However, this high ***K***_Mapp_ values may not actually reflect the activity range of the enzyme before saturation of the active site, due to the inhibition caused by high substrate concentrations, when it was the case. The substrate inhibition causes an artifact where the ***V***_max_ calculated is much higher than any activity measured, and the calculated ***V***_max app_ will never be reached. Correspondingly, the ***K***_mapp_, as the enzyme begins to be inhibited before the catalytic site is completely saturated (see the values of ***K***_mapp_ and ***K***_i_), does not correspond to the substrate concentration where the enzyme shows 50% of its maximal rate.

The inhibition by high substrate concentrations can be explained by transglycosylation reactions, by the binding of a second molecule of the substrate in the active site, or even by an osmotic effect (high substrate concentration) leading to a decrease in the reaction rate. Many insect α-glucosidases are classified in family 13 of glycosyl-hydrolases (GH13) and such enzymes are known to catalyze the glycoside linkages by retaining the anomeric configuration of the substrate. This type of mechanism allows these enzymes to do transglycosylation (Chiba, 1997). It is noteworthy that similar kinetic behavior was described for the α-glucosidase of *Acyrthosiphon pisum* (Cristofoletti et al., 2003). In this report, the authors consider that transglycosylation might be an adaptation for the obtention of monosaccharides from sucrose without the increase in osmolarity, as a simple hydrolytic reaction of a 0.7 M sucrose solution (phloem concentration) might result in osmotic shock for the midgut epithelial cells. In this respect, *L. longipalpis* enzymes might have the same biochemical adaptation. It would be very interesting to observe if this is a common trait of α-glucosidase from insects feeding on nectar or phloem sap, and verify if this is a case of evolutionary divergence or convergence.

Two main mechanisms of transglycosylation reactions using sucrose as substrate have been described. In the first, after the hydrolysis of the glycosidic bond, the glucosyl residue is retained in an intermediate linked to one of the enzyme catalytic carboxylates, and the fructose residue is released from the active site. After that, the glucosyl moiety is transferred to another sucrose molecule, resulting in a trisaccharide with two glucoses. After several rounds of this reaction, we expect the preferential release of the fructose and the generation of polyglucose chains linked to the initial sucrose molecule (Trincone, 2011). The second known mechanism for transglycosylation with sucrose as donor and acceptor consists in the retention of the fructosyl moiety as an intermediate linked to the catalytic glutamate, with the release of a glucose molecule after the initial hydrolysis of the glycosidic bond. After that, the fructosyl is transferred to an entering sucrose molecule, resulting in a trisaccharide with two fructoses. After several rounds, we expect releasing of most glucose from the substrate, and production of a polyfructose linked to an initial sucrose molecule (Han et al., 2015).

Our data strongly suggest that, in the case of transglycosylation in the presence of high sucrose concentrations, the mechanism preferred by *L. longipalpis* α-glucosidases is the first one described above. The first evidence that points to that is the avidity and preference of the female sand fly for fructose over glucose, which might be coherent with the release of fructose and a correspondent absorption system able to absorb this sugar. The second is that our activity measurements are based on the measurement of released glucose, and the release of glucose is hindered mainly by the first transglycosylation mechanism, as in the second type of transglycosylation this monosaccharide is released after the first hydrolysis step. A similar mechanism was proposed for the *A. pisum* enzyme (Cristofoletti et al., 2003), suggesting that this property may be present in a common ancestor of Eumetabola. Furthermore, it is important to note that additional experiments to confirm whether the inhibition by high substrate concentration was caused by transglycosylation and which mechanism is involved, are necessary.

Besides that, the high ***K***_mapp_ values and inhibition by substrate described for some enzymes of *L. longipalpis* in sucrose hydrolysis might be related to their involvement in metabolic control. Different enzymes are known to have a high ***K***_m_ value as a form of regulation of specific metabolic pathways. As the concentration of sucrose on sap is about 0.5–1.2 M (Gontijo et al., 1996), it could saturate the enzymes involved in sugar digestion, but with a high ***K***_mapp_ value these enzymes will need higher amounts of substrate (sucrose) to reach their maximum activity. This effect is increased by substrate inhibition, and so the digestion of sugars will occur at a lower rate than would occur if the ***K***_mapp_ were low. In this way, the availability of glucose for cell uptake might be strictly controlled.

Kinetic parameters for some α-glucosidases from insects have been determined using different substrates and their ***K***_m_ values show a lot of variation. *L. longipalpis* α-glucosidase from midgut tissue showed the highest ***K***_mapp_ when compared to others insects. Souza-Neto et al. (2007) described different isoforms of α-glucosidases from *Anopheles aquasalis* with a ***K***_m_ variation of 1.31–8.26 mM for the substrate pNPαGlu. The α-glucosidase III from *Apis mellifera* shows a ***K*m** value of 30 mM for sucrose and 13 mM for pNPαGlu (Nishimoto et al., 2001) and the α-glucosidase I from *Apis cerana japonica* 2.4 mM for sucrose (Wongchawalit et al., 2006).

The profiles obtained after gel filtration chromatography, especially for the midgut contents, and the biochemical parameters determined in different conditions, suggest the presence of different α-glycosidases acting in the metabolism of sand flies (Figure 7). We demonstrated at least three different α-glycosidases/sucrases that are secreted as soluble enzymes, and a major membrane-bound activity in the midgut of *L. longipalpis* females. To distinguish the specific roles of each of these α-glucosidases in the digestive process, purification and additional characterization are desired to understand how this enzymatic complex acts under different conditions and if the purified enzymes have different specificities or act sinergycally in the hydrolysis of substrates during digestion.

The compartmentalization of the insect digestive process is a hallmark of the understanding of insect physiology that was obtained during the XX century. According to that, in several insects the digestive process is divided among three basic compartments: the endoperitrophic lumen, the ectoperitrophic space, and the surface of the enterocytes (Terra and Ferreira, 2005). In this respect, glycoside hydrolases that act on the initial steps of digestion tend to be soluble enzymes, acting on large molecules as polysaccharides inside the endoperitrophic space. The most common enzymes related to the initial steps of carbohydrate digestion are amylases, cellulases, β-1,3-glucanases, xylanases, pectinases, chitinases, and lysozymes (Terra and Ferreira, 2005). Conversely, glycoside hydrolases acting in the intermediate and final stages of carbohydrate digestion, namely the hydrolysis of oligo- and disaccharides, respectively, tend to be confined in the ectoperithrophic space or bound to the apical cellular membrane of the enterocytes, as proteins anchored to the lipid bilayer or associated to the glycocalyx. In this category, we found α-glucosidases, β-glucosidases, β-galactosidases, trehalases, acetyl-hexosaminidases, β-fructosidases, and α-galactosidases (Terra and Ferreira, 2005).

Dipterans are considered as an advanced order in respect to the evolutionary acquisition of derived traits in the midgut, with the description of a well-compartmentalized distribution of enzymes related to the digestive process (Terra and Ferreira, 1994, 2005). In this respect, the presence of soluble α-glucosidases, secreted to the lumen and induced preferentially by blood, suggest that these enzymes might be acting on a large substrate molecule, during the initial stages of digestion. As the blood has insignificant amounts of polysaccharides, and α-glucosidases have low activity against this type of substrate, it is possible that the natural substrate of *L. longipalpis* soluble α-glucosidases consists in the carbohydrate moiety of blood glycoproteins or glycolipids. The tissue bound enzyme, which is induced by the ingestion of sucrose, might be participating in the canonical role of membrane bound glucosidases, the final digestion of small sugars as sucrose and other disaccharides. In this respect, the involvement of *L. longipalpis* adult α-glucosidases in physically separated stages of hydrolysis of very different food molecules might be the physiological and evolutionary basis for the diversification of this type of digestive enzyme activity.

These results together show that there are different α-glucosidases involved in the energetic metabolism of *L. longipalpis* females. Some of these enzymes might be specialized and some might act synergically in the hydrolysis of different substrates, being induced according to the feeding regime. Some of these α-glucosidases appear to be specialized in the sugar digestion, some may participate in the digestion of blood glycoproteins or glycolipids, and some might be involved in the mobilization of energetic storages during starvation. Our work increases the knowledge about the biology of phlebotomines, providing a detailed description of the induction of α-glucosidase activity in different physiological conditions, demonstrating the presence and especialyzation of α-glucosidases in different compartments of adult females of *L. longipalpis*.

## Author Contributions

SdC, PB, RD, and FG conceptualized and designed the work, and wrote and revised the manuscript. SdC obtained the experimental data. SdC and FG analyzed the data. All authors read and approved the final version.

## Conflict of Interest Statement

The authors declare that the research was conducted in the absence of any commercial or financial relationships that could be construed as a potential conflict of interest.

## Abbreviations

AMPSO: *N*-(1,1-dimethyl-2-hydroxyethyl)-3-amino-2-hydroxypropanesulfonic acid
BCA: bicinchoninic acid
CAPS: 3-(cyclohexylamino)-1-propanesulfonic acid
E64: (2S,3S)-3-(*N*-{(S)-1-[*N*-(4-guanidinobutyl)carbamoyl]3-methylbutyl}carbamoyl)oxirane-2-carboxylic acid
EPPS: 4-(2-hydroxyethyl)-1 piperazinepropanesulfonic acid
MOPS: 4 morpholinepropanesulfonic acid
MUαGlu: methylumbelliferyl-α-glucopyranoside
PMSF: phenylmethylsulfonyl fluoride
pNPαGlu: 4-nitrophenyl α-D-glucopyranoside.
