# Supplementary material for: Characterization of α-Glucosidases From Lutzomyia longipalpis Reveals Independent Hydrolysis Systems for Plant or Blood Sugars
Source: Front Physiol. 2019 Apr 10;10:248. doi: 10.3389/fphys.2019.00248 (PMC6468571; doi:10.3389/fphys.2019.00248)
Supplement: Supplementary file 2 [file Presentation_2.PPTX]

## Slide 1
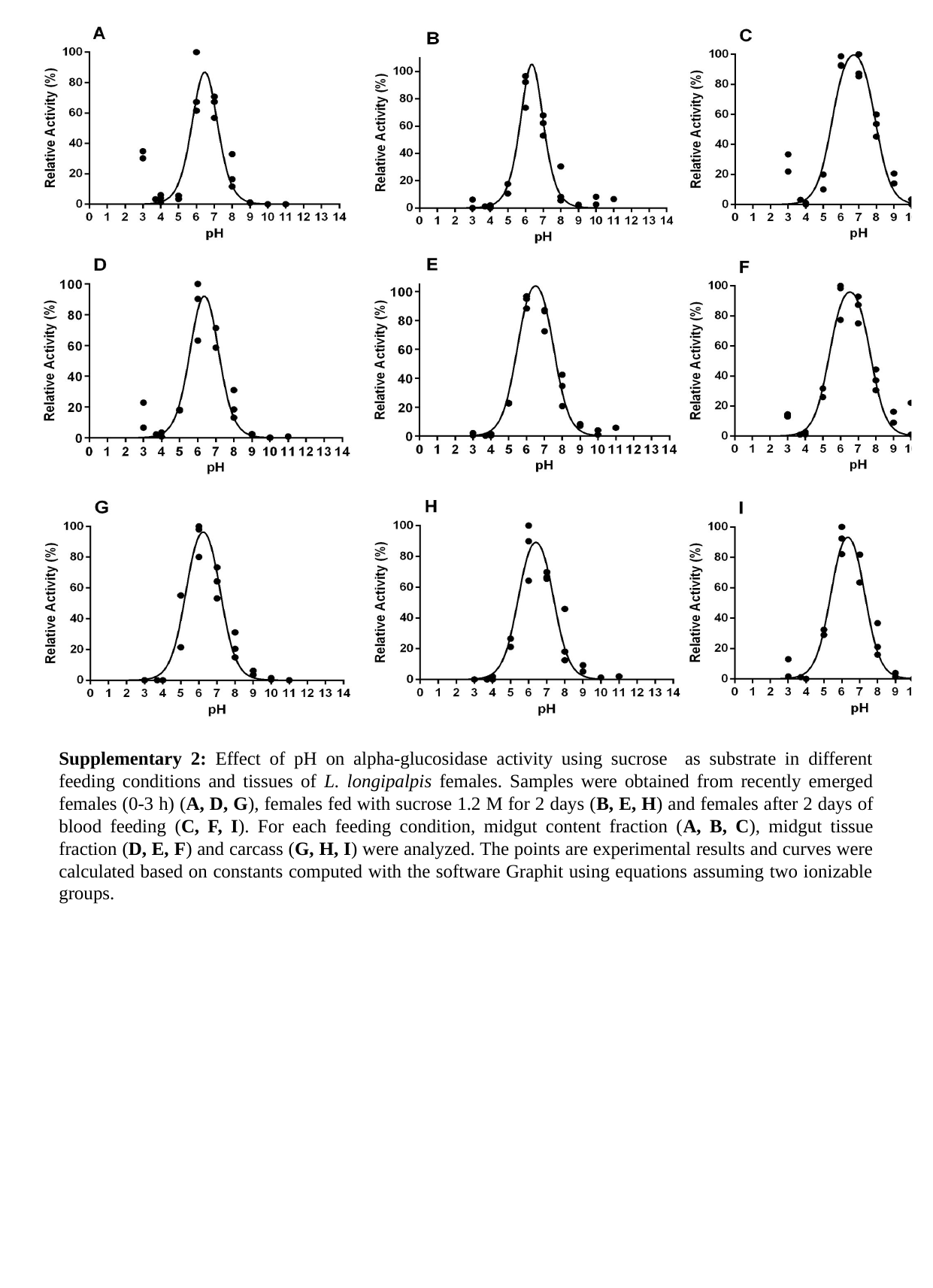

Supplementary 2: Effect of pH on alpha-glucosidase activity using sucrose as substrate in different feeding conditions and tissues of L. longipalpis females. Samples were obtained from recently emerged females (0-3 h) (A, D, G), females fed with sucrose 1.2 M for 2 days (B, E, H) and females after 2 days of blood feeding (C, F, I). For each feeding condition, midgut content fraction (A, B, C), midgut tissue fraction (D, E, F) and carcass (G, H, I) were analyzed. The points are experimental results and curves were calculated based on constants computed with the software Graphit using equations assuming two ionizable groups.
